# Recent progress of the tumor microenvironmental metabolism in cervical cancer radioresistance

**DOI:** 10.3389/fonc.2022.999643

**Published:** 2022-10-12

**Authors:** Junying Zhou, Ningjing Lei, Wanjia Tian, Ruixia Guo, Mengyu Chen, Luojie Qiu, Fengling Wu, Yong Li, Lei Chang

**Affiliations:** ^1^ Department of Obstetrics and Gynecology, The First Affiliated Hospital of Zhengzhou University, Zhengzhou, China; ^2^ School of Basic Medical Sciences, Zhengzhou University, Zhengzhou, China; ^3^ Cancer Care Centre, St George Hospital, Kogarah, NSW, Australia; ^4^ St George and Sutherland Clinical Campuses, School of Clinical Medicine, University of New South Wales (UNSW) Sydney, Kensington, NSW, Australia

**Keywords:** radiotherapy, radioresistance, metabolism, tumor microenvironment, cervical cancer

## Abstract

Radiotherapy is widely used as an indispensable treatment option for cervical cancer patients. However, radioresistance always occurs and has become a big obstacle to treatment efficacy. The reason for radioresistance is mainly attributed to the high repair ability of tumor cells that overcome the DNA damage caused by radiotherapy, and the increased self-healing ability of cancer stem cells (CSCs). Accumulating findings have demonstrated that the tumor microenvironment (TME) is closely related to cervical cancer radioresistance in many aspects, especially in the metabolic processes. In this review, we discuss radiotherapy in cervical cancer radioresistance, and focus on recent research progress of the TME metabolism that affects radioresistance in cervical cancer. Understanding the mechanism of metabolism in cervical cancer radioresistance may help identify useful therapeutic targets for developing novel therapy, overcome radioresistance and improve the efficacy of radiotherapy in clinics and quality of life of patients.

## 1 Introduction

Cervical cancer is the fourth most common malignant tumor and an important cause of death in women (1). There is a high incidence of cervical cancer in low-income and middle-income countries due to the low popularity of human papillomavirus (HPV) vaccination and cervical cancer screening (1–4). In all cervical cancer, squamous cell carcinoma accounts for 70%, followed by adenocarcinoma accounting for 20% (5). The standard therapies for cervical cancer include surgery, radiotherapy (RT), chemotherapy, and immunotherapy (6). For early-stage cervical cancer, radical surgery is the best option (7). For patients with locally advanced cervical cancer (LACC), concurrent chemoradiotherapy with radical surgery is the standard treatment (8). If patients have recurrent or metastatic cervical cancer, the standard treatment is chemoradiotherapy combined with immunotherapy after surgery, however the prognosis is still very poor (9, 10).

RT is the main treatment choice for LACC (11, 12). It directly causes tumor cell DNA double-strand breaks (DSBs), and the reactive oxygen species (ROS) induced by RT also indirectly causes DNA damage. At the cellular level, radiation-induced DNA damage and DSBs are fatal to cells. However, if the ability of DNA damage repair exceeds DNA damage speed, tumor cells will escape the effect of RT and lead to radioresistance (13–15). Although RT improves the therapeutic effect of cervical cancer patients greatly, the occurrence of radioresistance is still the main challenge for treatment failure (16, 17). Therefore, it is necessary to study the mechanism of radioresistance to overcome this problem and improve the efficacy of RT.

Studies have shown that tumor microenvironment (TME) plays an important role in radioresistance of cervical cancer (12). For instance, hypoxic TME enhanced radioresistance of cervical cancer cells by upregulating hypoxia-inducible factor 1α (HIF-1α) expression (12, 18). HIF-1α knockdown was found to enhance the radiosensitivity of Hela cervical cancer cells (19). In addition, metabolic reprogramming in TME was also reported to affect the efficiency of RT and contribute to cervical cancer radioresistance (20). For example, studies have shown that inhibition of glycolysis and lactic acid production improved the response of cervical cancer cells to single dose RT in ME180 cervical cancer cells (20). Thus, glucose metabolism determines the effect of RT, and metabolic reprogramming provides the key information for clinical cancer treatment. Other studies also suggest that metabolism reprogramming increased radioresistance of cancers (21–23). It was reported that STAT1 upregulated the key glycolytic enzymes lactate dehydrogenase A (LDHA) and pyruvate kinase type M2 (PKM2), promoted the productivity of glycolysis, and then enhanced the resistance to RT in SCC61 human squamous cell carcinoma (23). Serine/threonine kinase AKT mediated enhancement of aerobic glycolysis was reported to promote radioresistance of Hela cervical cancer cells (24). Thus, metabolic reprogramming is a very important factor in cervical cancer radioresistance and is worth of paying more attention.

This review discusses the complicated radioresistant mechanism of cervical cancer and focuses on the recent progress of TME metabolisms on radioresistance. The new research perspectives in this field are able to provide novel ideas and insights to overcome cervical cancer radioresistance with the development of novel targeted therapy.

## 2 Radiotherapy in cervical cancer treatment and the occurrence of radioresistance

Chemoradiotherapy is the main non-surgical treatment for patients with LACC (25), suggesting that RT is an important aspect in cervical cancer therapy especially in its late stages (25, 26). The main role of RT is demonstrated to destroy cancer cells and shrink tumor sizes, which is applied for postoperative adjuvant therapy. Fractional RT is usually used to treat cancer patients, generally 1.8 ~ 2.0 Gy per day and 5 days per week, for a cycle (26). Different types of cancer RT programs are not the same, and their responses to RT are also different (27).

Various mechanisms related to how RT kills tumor cells have been widely studied. For instance, ionizing radiation can penetrate tissues, destroy chemical bonds, and remove electrons from atoms to treat cancer (28). It also causes chromosomal mutations in cells, leading to cell death (29). Another mechanism that may be the most important one is the DSB of cancer cells after RT (30). In addition, ROS produced by RT indirectly induces cancer cell death (26). Superoxide anion (O^2 -^), hydrogen peroxide (H_2_O_2_) and hydroxyl radical (OH^-^) are the most common ROS (31, 32), which are effective molecules in RT. These ROS show high reactivity to a variety of cellular macromolecules including DNA, lipids and proteins, and induce biological changes, resulting in tumor cell death (28). Apoptosis, autophagy and necrotic cell death are the common forms of cancer cell death induced by ROS (33). Several clinical treatments, such as photodynamic therapy (PDT), whose mechanism mainly induces ROS to destroy and kill cervical cancer cells (34, 35).

**Figure 1:**
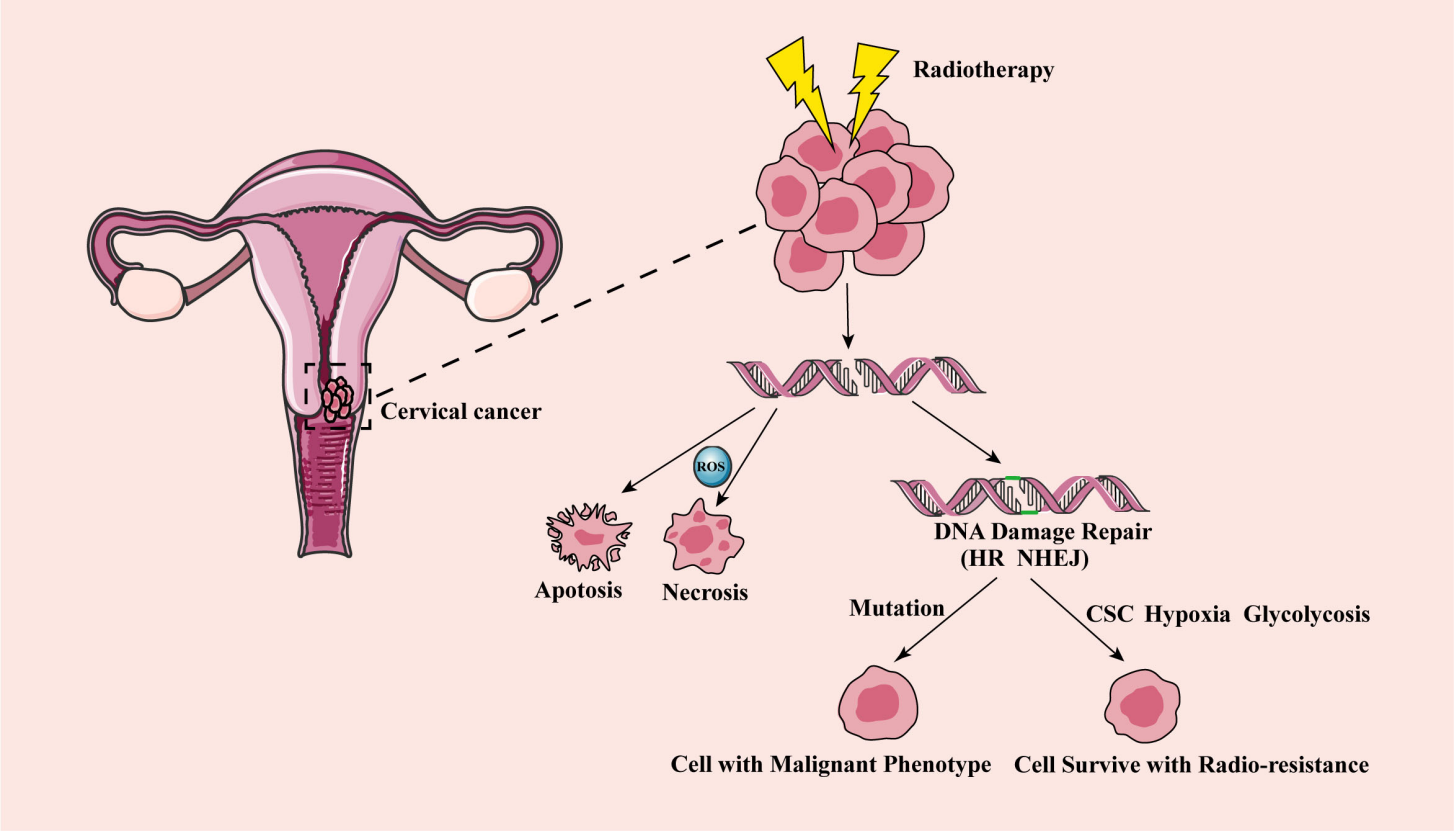
The mechanism of cervical cancer radioresistance after radiotherapy. After RT, DNA double strand breaks in cervical cancer cells, and some cancer cells show apoptosis and necrosis, reaching the effect of RT. Other fraction of cancer cells has DNA damage repair (including HR and NHEJ). The wrong repair path leads to the accumulation of acquired mutations, and tumor aggressivity and recurrence, while the correct repair leads to cell survival and radioresistance. CSC, cancer stem cell; HR, homologous recombination; NHEJ, non-homologous end joining; RT, radiotherapy.

Inhibition of DNA repair has been proved to be a method to improve radiosensitivity of cervical cancer (25). After RT, human DNA repair includes two important pathways homologous recombination (HR) and non-homologous end joining (NHEJ) (30, 36). The HR repair pathway is a precise form of repair that uses undamaged DNA sequences as a template to function in the S and G2 phases of cell cycle (37). Whereas the NHEJ pathway is an error-prone mechanism. It connects broken double-stranded DNA at all stages of the cell cycle, which may cause chromosomal connection errors, such as misalignment or ectopic position (29). DNA repair pathways regulated by HIF-1α, and HR and NHEJ pathways were reported to be inhibited due to hypoxia in breast cancer cells (38). In addition, under hypoxia, the expression of the NHEJ pathway related genes was also found inhibited, resulting in enhanced sensitivity of prostate cancer cells to RT (39). Furthermore, damaged NHEJ was demonstrated to lead to genomic instability, increase the proportion of acquired mutations or translocations, and induce tumorigenesis in leukemia (40, 41). Therefore, several studies have indicated that the key downstream factors in the HR and NHEJ repair pathways may become potential targets for enhancing the sensitivity of RT (42–46). For instance, RAD51 is a highly conserved protein that catalyzes DNA repair through HR repair pathway and modulates the sensitivity of cells to RT (47, 48). Inhibiting RAD51 was found to enhance the radiosensitivity of cervical cancer cells (47, 49, 50). Wang et al. showed that Sulforaphane (SFN) enhanced the radiosensitivity of cervical cancer cells by blocking RAD51 recruitment to the site of injury (49). Other studies also found that Ku70 and Ku80 proteins were involved in the repair of DSBs through NHEJ of DNA strands (51, 52). Cervical cancer cells with the low expression of Ku70 had higher radiosensitivity, and the survival rate of cervical cancer patients with low expression of Ku70 were also higher (53). It was reported that compared with Ku80 positive cervical cancer patients, Ku80 negative patients had a stronger response to RT. Inhibition of Ku80 was found to improve radiosensitivity, which has been confirmed in cervical cancer patients (15, 54). MRE11 is the core of MRN (MRE11-RAD50-NBS1) complex and plays an important role in DNA damage sensing and repairing (15, 55). After DNA damage caused by ionizing radiation, MRE11 recognized and excised DNA DSB ends for further repair (55). RhoC was found to regulate MRE11-mediated DNA repair through Rock2 and regulate radioresistance of cervical cancer (56). NHEJ protein DNA-PKcs was found to be up-regulated in the residual tissues of cervical carcinoma after RT and inhibiting the activity of DNA-PKcs improved the radiosensitivity of cervical cancer (57–60). PARP1 had a high ability to sense DNA damage, and it participated in the repair of single strand broken DNA. PARP1 inhibitor upregulated the sensitivity of cervical cancer to radiation (61, 62). The potential mechanism of radioresistance after RT in cervical cancer is shown in **Figure 1**.

Several oncogenes or tumor suppressor genes were reported to be involved in the regulation of RT. For example, retinoblastoma protein-interacting zinc finger gene (RIZ), located on chromosome 1q36, is a tumor suppressor whose expression is downregulated in a variety of tumors (63, 64). It was found that cervical cancer cells overexpressing RIZ increased the apoptosis rate and DNA damage after RT compared to the control group (65). This study showed that RIZ improved the radiosensitivity of cervical cancer cells and was a potential therapeutic target of RT combined with gene therapy for cervical cancer patients. P53 is an important regulator of cell cycle and DNA repair. After the imbalance of p53 in cervical cancer cells, its regulatory function was confirmed, and the irradiated cells underwent uncontrolled DNA repair, leading to radioresistance (66). P73 has homology with the well-known tumor suppressor gene p53 and is considered as a new tumor suppressor gene. The high expression of p73 was significantly correlated with the radiosensitivity of cervical cancer and played an important role in promoting radiosensitivity of cervical cancer (67). XAV939 is an inhibitor of the Wnt/β-catenin signaling pathway. When combined with RT, XAV939 inhibited proliferation and increased apoptosis of human medulloblastoma cells (68). In cervical cancer, XAV939 promoted apoptosis induced by RT, holding a therapeutic potential in increasing clinical efficacy (69). Inhibiting the repair of DNA damage caused by RT sensitized the radiation response of cervical cancer (70). PXN gene was up regulated in cervical cancer cells, and its overexpression reduced the apoptosis rate after RT, leading to radioresistance (71). The DNA repair molecules and gene regulation cervical cancer are summarized in **Table 1**.

**Table 1:** List of DNA repair molecules and gene regulation in cervical cancer after radiotherapy.

Gene	Function	DNA Repair path	Reference
RAD 51	DNA damage repair	HR	(47–50)
Ku 70	DNA double strand break identification and repair	NHEJ	(53)
Ku 80	DNA double strand break identification and repair	NHEJ	(15, 54)
MRN	DNA double strand break repair	NHEJ	(56)
DNA-PKcs	Add Poly ADP-Ribose to the damaged site of single stranded DNA to promote DNA repair.	NHEJ	(57–60)
PARP1	DNA single-strand breaks repair	NHEJ	(61, 62)
RIZ	Induce apoptosis and DNA damage	/	(65)
P73	Induce cell arrest and apoptosis	/	(67)
XAV939	Inhibit WNT signaling pathway and promote apoptosis	/	(69)
P53	Regulate cell cycle and DNA repair	/	(66)
PXN	Upregulation of bcl-2 and inhibition of apoptosis	/	(71)

MRN, MRE11-RAD50-NBS1 complex; DNA-PKcs, DNA-dependent protein kinase; PARP1, Poly (ADP-ribose) polymerase-1; RIZ, retinoblastoma protein-interacting zinc finger gene; HR, homologous recombination; NHEJ, non-homologous end joining.

Inhibitors of DNA repair pathway are crucial to improve the therapeutic efficiency of patients. Some small molecule inhibitors have been used in clinical trials or applied in clinical practice (72). DNA-PK is a key driver of NHEJ pathway, and Pepostertib is an inhibitor of DNA-PK, which destroys DNA repair by inhibiting the activity of DNA-PK to improve the therapeutic effect (73). In one preclinical study, AZD7648 (an effective and specific DNA-PK inhibitor) combined with RT induced tumor regression in mice and improved the therapeutic effect (74). X-ray repair cross complement protein 5 (XRCC5) is an ATP dependent DNA helicase, which provides a start for the DNA repair mechanism of the NHEJ pathway when DNA double strand breaks. Targeting XRCC5 with Myrosin G was found to play a positive therapeutic role (75). CB-5083, as a specific small molecule inhibitor of p97, prevented the decomposition of MRN complex at the DNA damage site during ionizing radiation and damaged DNA repair, thus enhancing the killing function of tumor cells after RT (76). NHEJ inhibitors can be used in combination with standard cancer therapies to reduce therapeutic dose and improve clinical therapeutic effect. Orapanib, a PARP inhibitor, destroyed the localization of base excision repair effector XRCC1 and NHEJ proteins Ku80 and XRCC4, enhanced the sensitivity of cervical cancer cells to cisplatin, demonstrating the potential of PARP inhibitors in the treatment of cervical cancer (77). Santu et al. found that the PARP inhibitor Rucaprib is the most effective radiosensitizer for cervical cancer, which improved the effect of RT for cervical cancer patients (78). The small molecule inhibitors associated with DNA repair are summarized in **Table 2**. Targeting RT related genes is in a good position to promote DNA damage and apoptosis induced by radiation, which provides a new strategy for cervical cancer RT.

**Table 2:** List of small molecule inhibitors about DNA repair.

Target	Inhibitor	Main Function	Reference
DNA-PK	Pepostertib	Destroy NHEJ and inhibit DNA repair	(73)
	AZD7648	Inhibit DNA double strand break repair	(74)
XRCC5	Myrosin G	Target XRCC5 and inhibit DNA repair	(75)
p97	CB-5083	Prevents the decomposition of MRN complex from DNA damage site	(76)
PARP	Orapanib	Destroying XRCC1 and NHEJ, aggravating S and G2/M block	(77)
	Rucaprib	Inhibition of DNA repair and radiosensitization	(78)
PI3K/mTOR	NVP-BEZ235	G1 cell cycle arrest and apoptosis induction	(79)
	1,3,5-triazine derivatives	G1 cell cycle arrest	(80)
mTOR	AZD8055	Inhibiting proliferation and glycolysis, inducing apoptosis	(81)

DNA-PKcs, DNA-dependent protein kinase; XRCC5, X-ray repair cross complement protein 5; PARP, Poly (ADP-ribose) polymerase; NHEJ, non-homologous end joining.

## 3 Tumor microenvironment contributes to of cervical cancer radioresistance

The presence of immune cells and hypoxia in TME is closely related to cervical cancer radioresistance. For example, the high level of tumor-associated neutrophils was reported to lead to radioresistance in cervical cancer cells and associated with a poor prognosis in patients (18). In addition, many studies have focused on the role of the hypoxic TME in promoting cervical cancer radioresistance (12). The cancer cells in TME usually contain high amount of antioxidants. High levels of glutathione (GSH) were found to boost radioresistance of cervical cancer cells by clearing ROS (82). Therefore, developing radiosensitizers based on reducing antioxidants may be a potential strategy for cervical cancer therapy.

Hypoxia is one of the characteristics of cervical cancer and reduces the effect of RT (83–85). The imbalance between tumor cell growth and neovascularization, as well as the abnormal morphology and function of tumor neovascularization leads to hypoxic microenvironment (86). Hypoxia was demonstrated to enhance the resistance to RT, resulting in a poor prognosis of cervical cancer patients (83, 84, 87).

RT plays an active role in the treatment of cervical cancer; however, this treatment also paradoxically leads to radioresistance through the changes of transcription factors in TME. Epithelial-mesenchymal transition (EMT) is closely related to the effect of RT. Twist is a key transcription factor of EMT, and its expression is positively correlated with hypoxia. Downregulation of Twist reversed the radioresistance induced by hypoxia and enhanced the sensitivity of cervical cancer cells to RT (85). In addition, upregulation of Twist promoted the localization of nuclear epidermal growth factor receptor (EGFR) and the expression of nuclear DNA-PKcs, and enhanced the repair of DNA damage caused by RT (85). Another study showed that Lcn2 interacted with HIF-1α to promote the formation of radioresistant phenotype in nasopharyngeal carcinoma. It was found when Lnc2 was downregulated, the ability of cell colony formation and DNA damage repair were significantly reduced, which indicates that inhibiting Lnc2 improved the effect of RT for nasopharyngeal carcinoma (88). Although RT works for cancer, it paradoxically promotes metastasis and enhances the invasion of cancer through EMT induced by RT (89). Ionizing radiation was reported to activate multiple EMT-induced transcription factors, including HIF-1, ZEB1 and STAT3 (26, 90–92), which activate the corresponding signaling pathways to enhance the EMT ability of tumor cells. In cervical cancer, it has been proved that the EMT induced by RT enhanced the viability and invasiveness of cancer cells (89). In addition, EMT-induced transcription factors endow cells with cancer stem cell (CSC) properties and promote the production of CSCs. CSC is recognized as a radioresistant cell, which plays a role mainly by reducing radiation-induced DNA damage and enhancing DNA repair ability (93).

Immune cells in TME also contribute to the progression of cervical cancer, which makes the immunotherapy an important treatment choice. HPV E6 and E7 proteins were found to promote the expression of programmed cell death protein 1 (PD-1) and programmed death-ligand 1 (PD-L1) in cervical cancer (94). It is widely known that PD-1 and PD-L1 upregulation suppresses the activation of T cells, resulting in immune escape of tumor cells (95). In June 2018, the US Food and Drug Administration (FDA) approved an anti-PD1 antibody pembrolizumab for the treatment of several types of cancer including recurrent or metastatic cervical cancer (96). Another important molecule in suppressing T cell activation is the cytotoxic T-lymphocyte–associated antigen-4 (CTLA-4), and it’s up-regulation leads to immune tolerance of cancer cells (97). PD-L1 blockade and CTLA-4 inhibitor ipilumumab were reported to attenuate tumor induced inhibitory signal transduction, stimulate T cell activation, and play an anti-tumor role (94).

Therefore, targeting TME components is promising for developing novel cervical cancer therapy to reduce radioresistance.

## 4 Metabolic factors affect cervical cancer radioresistance

The metabolic disorder of cancer cells is closely related to tumorigenesis and therapeutic effects (98). Metabolic reprogramming is regulated by abnormal activation of proto-oncogenes and loss of tumor suppressor genes (99). In addition, TME influences various aspects of the tumor metabolism, including glucose metabolism, amino acid metabolism and lipid metabolism, which may contribute to the modulation of cervical cancer radioresistance (100–102).

### 4.1 Glucose metabolism and cervical cancer radioresistance

Cervical cancer mainly obtains energy through aerobic glycolysis to maintain the growth and proliferation of cells. Blocking glycolysis inhibits the growth of cancer cells and induces apoptosis, which provides a new approach for the clinical treatment of cervical cancer (103). The process of aerobic glycolysis in cervical cancer cells is mediated by key enzymes such as glucose transporter 1 (GLUT1), LDHA, hexokinase 2 (HK2) and aldolase A (ALDOA), which enhance glucose uptake and lactate production, and promote the progression of cervical cancer (104–108). The Wnt/β-catenin pathway is upregulated in cancer and promotes the activation of Warburg Effect, including the enhancement of enzyme activity in the glycolytic pathway and the acceleration of glutamate decomposition, which in turn promotes the production of lactate. The high lactate microenvironment stimulates the expression of vascular endothelial growth factor (VEGF), promotes tumor angiogenesis and induces cell migration (109). PI3K/mTOR is a key signaling pathway for cell proliferation, and researchers have made great efforts to inhibit this pathway. Li et al. inhibited the glycolysis process of cervical cancer cells through the mTOR inhibitor AZD8055 to fight against the unlimited proliferation of tumor cells (81). Several groups also used NVP-BEZ235 and new 1,3,5-triazine derivatives to double block PI3K/mTOR signaling pathway to inhibit the proliferation of cervical cancer cells, so as to enhance the therapeutic response (79, 80).

**Figure 2:**
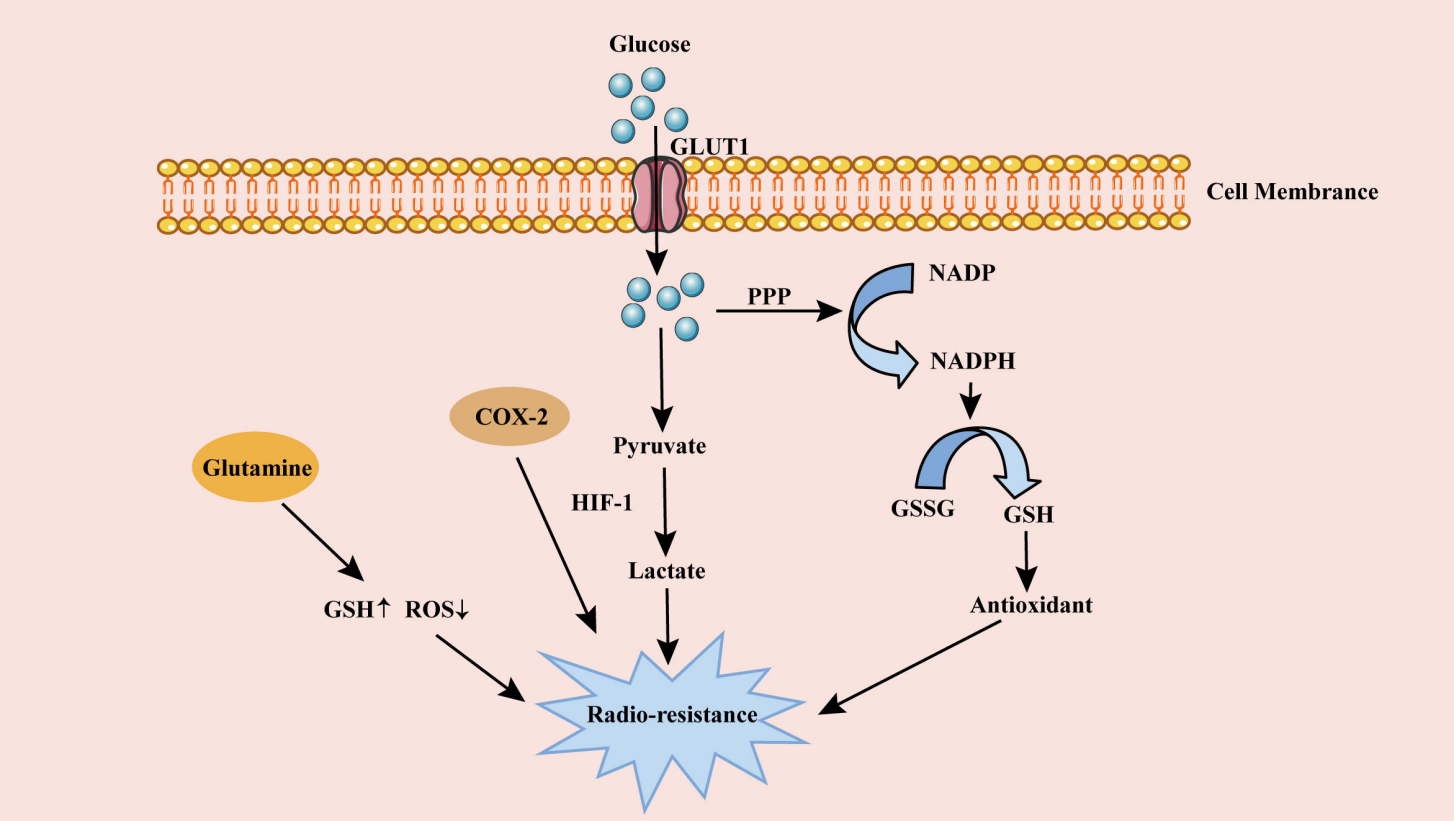
The role of metabolism on cervical cancer radioresistance. Glucose is transported into cells through GLUT1 for glycolysis and produces a large amount of lactic acid, enhancing cervical cancer radioresistance. Glucose in cells also produces GSH through PPP pathway. The antioxidant GSH weakens DSB after RT, resulting in radioresistance of cervical cancer cells. High expression of COX-2 in lipid metabolism and Glutamine in amino acid metabolism also contribute to cervical cancer radioresistance. GLUT1, glucose transporter 1; PPP, pentose phosphate pathway; ROS, reactive oxygen species; HIF-1, hypoxia-inducible factor 1; GSSG, oxidized glutathione; GSH, glutathione; RT, radiotherapy.

Cancer cells choose aerobic glycolysis with less capacity rather than aerobic oxidation with more capacity, which seems to be an uneconomical way. However, cancer cells upregulate GLUT and produce ATP with high efficiency, in order to meet their high energy needs (107). In addition, the metabolic mode of aerobic glycolysis also meets the proliferation needs of cancer cells. Glycolysis produces many metabolic intermediates and precursors, which enter various biosynthetic pathways, such as the pentose phosphate pathway (PPP), to generate amino acids and nucleic acids, and then synthesize biological macromolecules and organelles required for cell proliferation (110, 111). This metabolic mode produces pyruvate, lactic acid, NADPH and hydrogen ions. NADPH converts oxidized glutathione (GSSG) to glutathione (GSH) (**Figure 2**). These products and glutathione up-regulate the endogenous antioxidant capacity of cells, thereby directly or indirectly working for antagonistic RT (112).

#### 4.1.1 GLUT1

GLUT is a family of carrier proteins embedded in the cell membrane to transport glucose, which is widely distributed in various tissues in the body. The distribution of GLUT *in vivo* and its affinity with glucose molecules are significantly different. Among the 14 glucose transporters, GLUT1 is the most common and widely distributed one (21, 113). It is a human unidirectional protein encoded by SLC2A1 gene that is important for glucose uptake (114). It is up regulated in many types of cancer and metabolic diseases, and involved in the disease progression (115, 116). Inhibition of GLUT1 was found to downregulate glycolysis and inhibited the growth of cervical cancer cells *in vitro* and *in vivo* (117). Compared with normal cervical epithelium, the expression of GLUT1 is increased in cervical cancer and is associated with lymphatic metastasis (118).

There are different views on the role of GLUT1 in cervical cancer prognosis. Several lines of evidence suggest that high expression of GLUT1 indicates a poor prognosis of cervical cancer (105, 106, 119–121). However, another study suggested that the expression of GLUT1 was not correlated with the prognosis of cervical cancer (122, 123). This contradict requires more clinical studies to explore the role of GLUT1 in the prognosis of cervical cancer.

It was reported that high expression of GLUT1 in cervical cancer more likely leads to radioresistance (124). In a prospective study, it was found that GLUT1 could be used as a biomarker of cervical cancer radioresistance for individualized treatment (125). Pierre Benoit Ancey et al. found that the loss of GLUT1 in tumor-related neutrophils enhanced the effect of RT in patients with lung cancer. The potential mechanism may be that the lack of GLUT1 weakens the direct killing effect of neutrophils on cancer cells (126). In addition, increased GLUT1 expression was significantly associated with radioresistance in rectal cancer, oral cancer and esophageal cancer (127–130). Therefore, inhibiting the expression of GLUT1 may be used as a new potential therapeutic target to improve RT sensitivity of cancer patients (125).

#### 4.1.2 LDHA

LDHA is a kind of NAD dependent kinase, which is a homologous or heterotetramer molecule mainly located in cytoplasm. LDHA plays a key role in glycolysis to convert pyruvate to lactate and convert NADH to NAD (131). LDHA is up regulated in many cancers, resulting in increased catalytic product lactate and poor prognosis (121, 132–134). High lactic acid forms an acidic TME, promotes tumor to acquire an aggressive phenotype, and increases the risk of tumor metastasis and recurrence (135). It was reported inhibition of LDHA reduced the proliferation and invasion of cervical cancer cells (136, 137).

High lactate level promotes radioresistance of malignant tumor cells. A study on chemoradiotherapy resistance in advanced cervical cancer showed that the expression of LDHA was up-regulated in chemoradiotherapy resistant group by microarray analysis compared to chemoradiotherapy sensitive group (138). Another study also found that a relatively high expression level of LDHA in cervical cancer cells was resistant to RT, which helped cancer cells carry out aerobic glycolysis and promoted radioresistance (139). Therefore, LDHA inhibitors may be a promising strategy to improve the effect of RT and provide a certain foundation for the new drug development (140, 141).

#### 4.1.3 PKM2

PK is another key enzyme in glycolysis, which catalyzes the conversion of phosphoenolpyruvate and adenosine diphosphate to pyruvate that is the last irreversible conversion process of glycolysis (142–144). The M2 subtype PKM2 is highly expressed in many cancers, playing an important role in maintaining the metabolism of cancer cells (143). The high expression level of PKM2 was used as a marker to predict the cervical cancer prognosis (145). One study showed that PKM2 knockdown inhibited EMT *via* the Wnt/β-catenin pathway, thereby suppressing cervical cancer cell proliferation and invasion (146). Another study reported that knockdown of PKM2 in SiHa and HeLa cervical cancer cells promoted DNA DSBs leading to RT sensitivity (147). In addition, PKM2 knockdown leads to G2/M cell cycle arrest, and the expression of CSC marker NANOG decreases, which significantly enhances the RT effect of cervical cancer cells (147). Zhao et al. found that the high expression of PKM2 was related to the poor prognosis of locally advanced cervical squamous cell carcinoma and contributed to the generation of radioresistance using multivariate COX regression analysis (148). Therefore, targeting PKM2 is a new strategy to improve the RT effect for cervical cancer.

#### 4.1.4 HK2

Glucose can be converted into glucose 6-phosphate under the catalysis of HK2, which is the first step of glycolysis (149). Downregulation of HK2 inhibited glycolysis, reduced glucose consumption and lactate production of cells, and also inhibited cell proliferation and induced apoptosis (137, 150). Whether HK2 regulates the biological behavior and treatment of cancer cells through the glycolytic pathway needs to be further explored. Knockdown of HK2 in cervical cancer cells inhibited proliferation and migration and promoted cell apoptosis (151). Targeting HK2 was found to reduce the glycolysis of cervical cancer cells and enhance the sensitivity of cervical cancer to RT (152). It was showed that increased expression of long non-coding RNA urothelial cancer associated-1 (lncRNA UCA1) by RT enhanced glycolysis by targeting HK2, thereby promoting cervical cancer radioresistance (153). In the established radioresistant cervical cell lines SiHa and HeLa, it was also found that lncRNA UCA1 promoted radioresistance of the cells through enhancement of HK2 and glycolysis (153). Similarly, in the study of locally advanced cervical squamous cell carcinoma, it was found that the high level of HK2 was one of the risk prognostic factors for cervical cancer patients, and associated with the low radiosensitivity (154). Inhibiting HK2 to reduce the dependence of cervical cancer cells on glycolysis is a potential strategy to promote cervical cancer RT.

The glycolysis related proteins in cervical cancer radioresistance are summarized in **Table 3**.

**Table 3:** List of glycolysis related proteins in cervical cancer radioresistance.

Metabolism Enzyme	Canonical Function	Mechanism of Radioresistance	Reference
GLUT 1	Glucose transport	High aerobic glycolysis Lymph node metastasis, Endogenous markers of hypoxia	(117, 118, 124, 125)
LDHA	Convert pyruvate to lactate and NADH to NAD	Forming high lactic acid tumor microenvironment, High aerobic glycolysis	(139)
PKM2	Conversion phosphoenolpyruvate and adenosine diphosphate to pyruvate	Regulate cell cycle, Maintain stem cell characteristics.	(147)
HK2	Conversion of glucose to glucose 6-phosphate	Removal reactive oxygen species and free radicals, Promote glycolysis	(152)

GLUT1, glucose transporter 1; LDHA, dehydrogenase A; PKM2, pyruvate kinase type M2; HK2, hexokinase 2.

### 4.2 Lipid metabolism and cervical cancer radioresistance

Increased lipid uptake and storage in cancer cells contribute to tumor growth and proliferation. More and more evidence shows that lipid metabolism reprogramming plays a very important role in the development of cancer (155–157). The fatty acid is an important component of cell membrane structure, which maintains the fluidity of cell membrane. In case of metabolic emergency, fatty acid decomposition is also the main source of energy (158, 159). Targeting fatty acid metabolism was reported to improve the radiosensitivity of many cancers (160, 161).

In the treatment of cervical cancer patients, reasonable supplementation of high polyunsaturated fatty acids was demonstrated to improve the effect of RT (162). The lipid metabolites in high-resolution magic angle proton magnetic resonance spectroscopy spectrum was used to evaluate the apoptosis of cervical cancer (163). Ferulic acid was effectively combined with RT to increase lipid peroxide of cervical cancer cells and improved radiosensitivity (164). Cyclooxygenase (COX) converted arachidonic acid into prostaglandins, and COX-2 inhibitor phosphorylated p53 in irradiated cervical cancer cells, reducing the cervical cancer radioresistance (102). Currently, the study on lipid metabolism in cervical cancer radioresistance is very limited, but related studies in other types of cancer may give some insights for future investigation. For example, arachidonate 15-lipoxygenase deficiency was found to reduce DNA DSBs induced by RT and induce radioresistance in rectal cancer cells (165). Another study found that targeting fatty acid synthase enhanced the sensitivity of nasopharyngeal carcinoma to RT (161). In addition, targeting carnitine palmitoyl transferase 1 (CPT1A) mediated fatty acid oxidation also improved the effect of RT for nasopharyngeal carcinoma (166).

### 4.3 Amino acid metabolism and cervical cancer radioresistance

Cancer cells need to obtain enough amino acids for biosynthesis to adapt to the characteristics of rapid proliferation. Amino acid metabolism is closely related to the occurrence and development of cancer, and targeting amino acid metabolism provides a potential therapeutic strategy for cancer treatment (167).

The best-studied amino acid in this metabolic pathway is glutamine, which plays an important role by providing nitrogen and carbon in biosynthesis (168, 169). Studies have shown that glutamine metabolism was closely related to cancer radiosensitivity (170, 171). It has been found that in human cervical cancer samples, the content of phosphate-activated mitochondrial glutaminase2 (GLS2) in the radioresistant group was higher than that in the radiosensitive group. When GLS2 was knocked down, the level of antioxidant glutathione decreased and ROS increased after RT, which promoted the radiosensitivity of cervical cancer cells (172). In another study, the use of glutaminase inhibitors enhanced the radiosensitivity of cervical cancer (101). In addition, glutamine was decomposed by glutaminase, which enhanced the radiosensitivity of prostate cancer (173). Paradoxically, in HeLa cell culture, the addition of supraphysiologic glutamine concentration did not enhance the radioresistance of cervical cancer cells (174). Indepth study is needed about the effect of glutamine on cervical cancer RT. Aminopeptidase N (APN), as a transmembrane exopeptidase, is expressed at a level consistent with the increased malignant behavior of tumors (175). *In vitro* and *in vivo* studies showed that APN inhibitor ubenimex enhanced apoptosis and cell damage induced by RT, and improved the radiosensitivity of cervical cancer (176).

Currently, other amino acids regulating metabolism that affects cervical cancer radioresistance have not been reported yet, which requires further study.

## 5 Conclusions and perspectives

Radioresistance has become a major problem in the management of cervical cancer patients. This review summarizes the mechanism of radioresistance in cervical cancer from different aspects. Most scholars believe that the enhancement of DNA repair ability after RT leads to the reduction of cervical cancer cell death. In addition, EMT and CSCs induced by RT promote radioresistance, leading to cancer recurrence and metastasis.

Here, we focus on TME to investigate how to overcome the radioresistance and promote the efficiency of RT. Hypoxic TME not only directly reduces radiosensitivity due to the reduction of ROS, but also indirectly reduces radiosensitivity by promoting glycolysis. Glucose metabolism is a very important factor in occurrence of radioresistance in cervical cancer. The intermediate products produced by glycolysis are suitable to be used as the precursor of biosynthesis to promote the growth and proliferation of cervical cancer cells. Lactic acid produced by glycolysis promotes radioresistance of cervical cancer cells, and lactic acid inhibitors are expected to become new products to improve the effect of RT. Although the study of lipid metabolism and amino acid metabolism is very limited, we review the important findings related to cervical cancer. Glutamine has a relatively obvious effect on RT and low-level glutamine can increase the content of intracellular ROS to improve sensitivity of cervical cancer cells to RT. In addition, inhibiting the NHEJ pathway to block DNA repair makes tumor cells sensitive to RT, which is also a promising therapeutic strategy (40). In recent years, immunotherapy has become a novel promising treatment, and is used in combination with traditional chemotherapy or RT for advanced and recurrent cervical cancer patients. Anti-PD-1/PD-L1 and anti-CTLA-4 therapies are commonly used in cervical cancer immunotherapy to improve the prognosis of cervical cancer patients. Whether the metabolic programming can give insights for the development of immunotherapy to improve the current cervical cancer treatment is still unknown and worth of putting more efforts for further investigation.

There is no doubt that targeting the key enzymes and intermediates of metabolism is a promising strategy to improve the radiosensitivity of cervical cancer patients. With the in-depth study on the mechanisms related to metabolism with modern omics technologies, it is possible to develop personalized treatment plans to improve the current treatments and prognosis of cervical cancer patients with different metabolic status.

## Author contributions

JZ: draft the manuscript and iconography. WT, MC, RG, LQ, and FW: proofread the manuscript and iconography. LC, NL, YL, and JZ: conceptualized, supervised, and finalized the manuscript. All authors contributed to the article and approved the submitted version.

## Funding

This work was supported by Henan Provincial Medical Science and Technology Research Plan Joint Provincial and Ministry Youth Project (No.SB201902013), Scientific and Technological Project of Henan Province (No.182102310325 & 192102310069), Henan Province Colleges and Universities Innovative Talent Support Program (No.21HASTIT044), Henan Medical Education Research Project (No.Wjlx2020062), Henan Youth Talent Promotion Project (No.2020HYTP052), Key Research and Development Project of Henan Province (No.222102310539), Henan Province Young and Middle-aged Health Science and Technology Innovative Talent Training Project (No.YXKC2020039), Central Plains Youth Top Talent Project, and High-level Talents Return to China for Research Funding Project.

## Conflict of interest

The authors declare that the research was conducted in the absence of any commercial or financial relationships that could be construed as a potential conflict of interest.

## Publisher’s note

All claims expressed in this article are solely those of the authors and do not necessarily represent those of their affiliated organizations, or those of the publisher, the editors and the reviewers. Any product that may be evaluated in this article, or claim that may be made by its manufacturer, is not guaranteed or endorsed by the publisher.
